# Supplementary figures and images for: A P2P multi-path routing algorithm based on Skyline operator for data aggregation in IoMT environments
Source: PeerJ Comput Sci. 2023 Nov 22;9:e1682. doi: 10.7717/peerj-cs.1682 (PMC10702938; doi:10.7717/peerj-cs.1682)

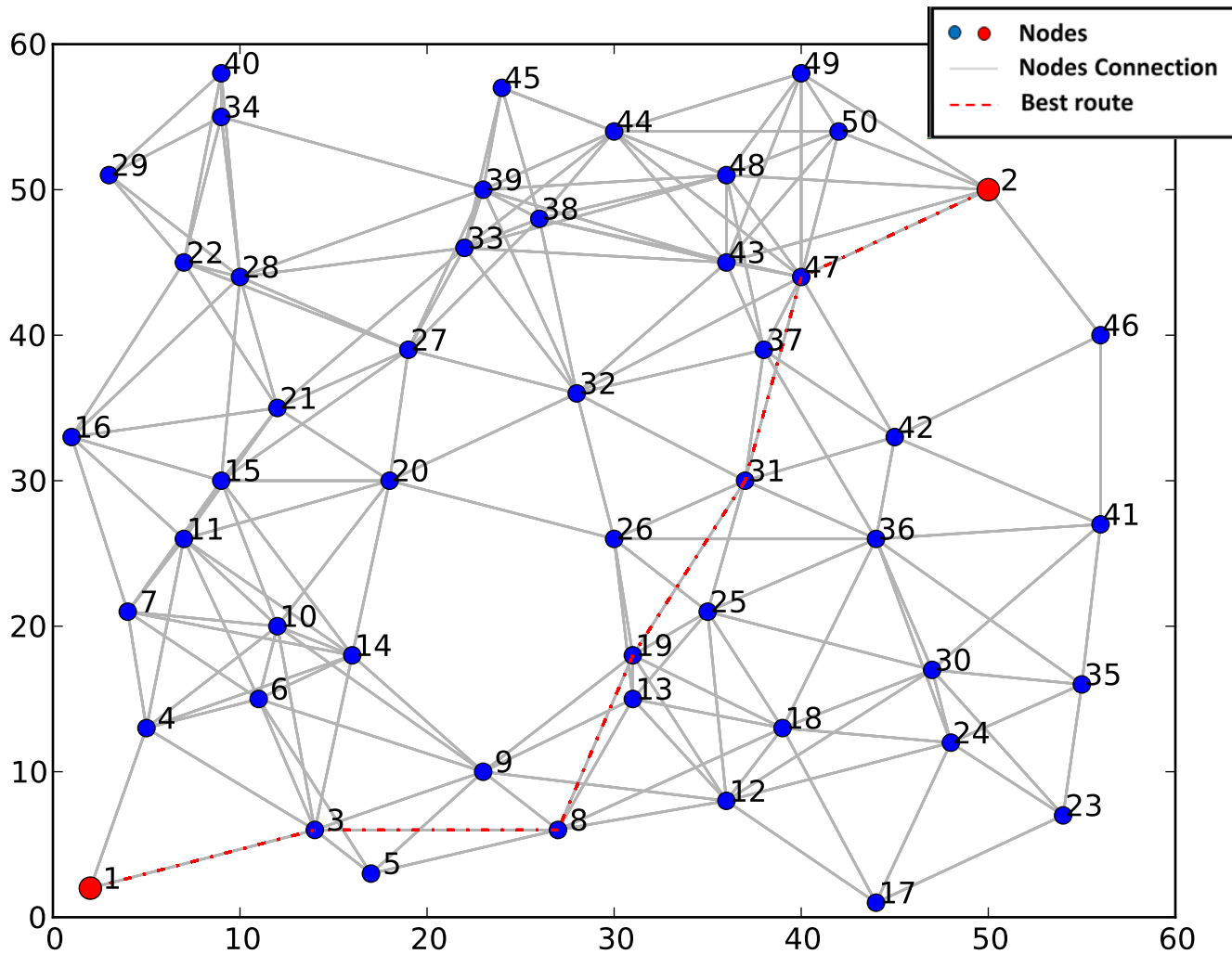

Supplement: Supplemental Information 1 — The code is written in NC programming language and is simulated using TOSSIM Simulator via Python script. The obtained values are available in the Excel files. [file peerj-cs-09-1682-s001.zip › Implementation/Simulation/Hop/results.pdf]

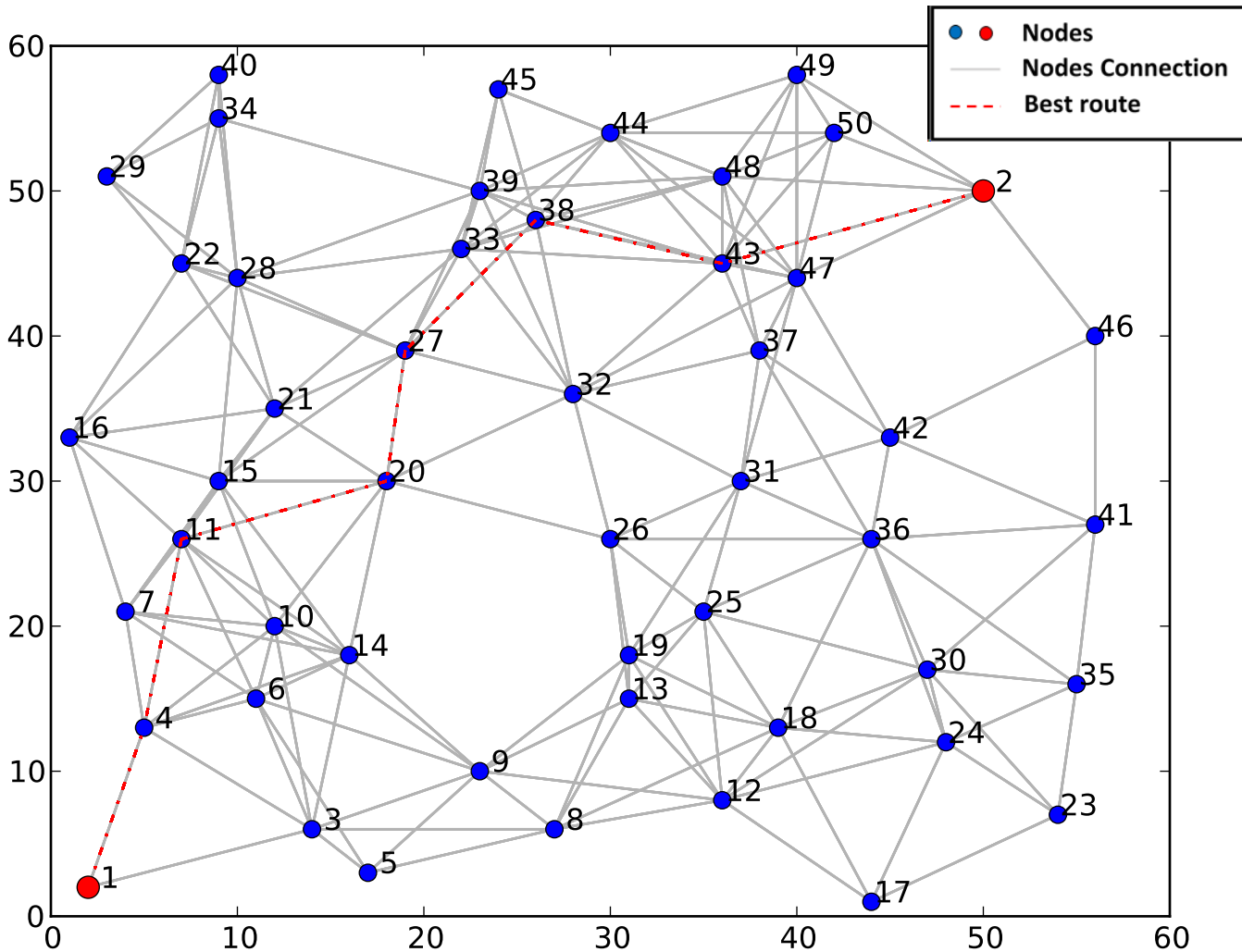

Supplement: Supplemental Information 1 — The code is written in NC programming language and is simulated using TOSSIM Simulator via Python script. The obtained values are available in the Excel files. [file peerj-cs-09-1682-s001.zip › Implementation/Simulation/Energy/results.pdf]

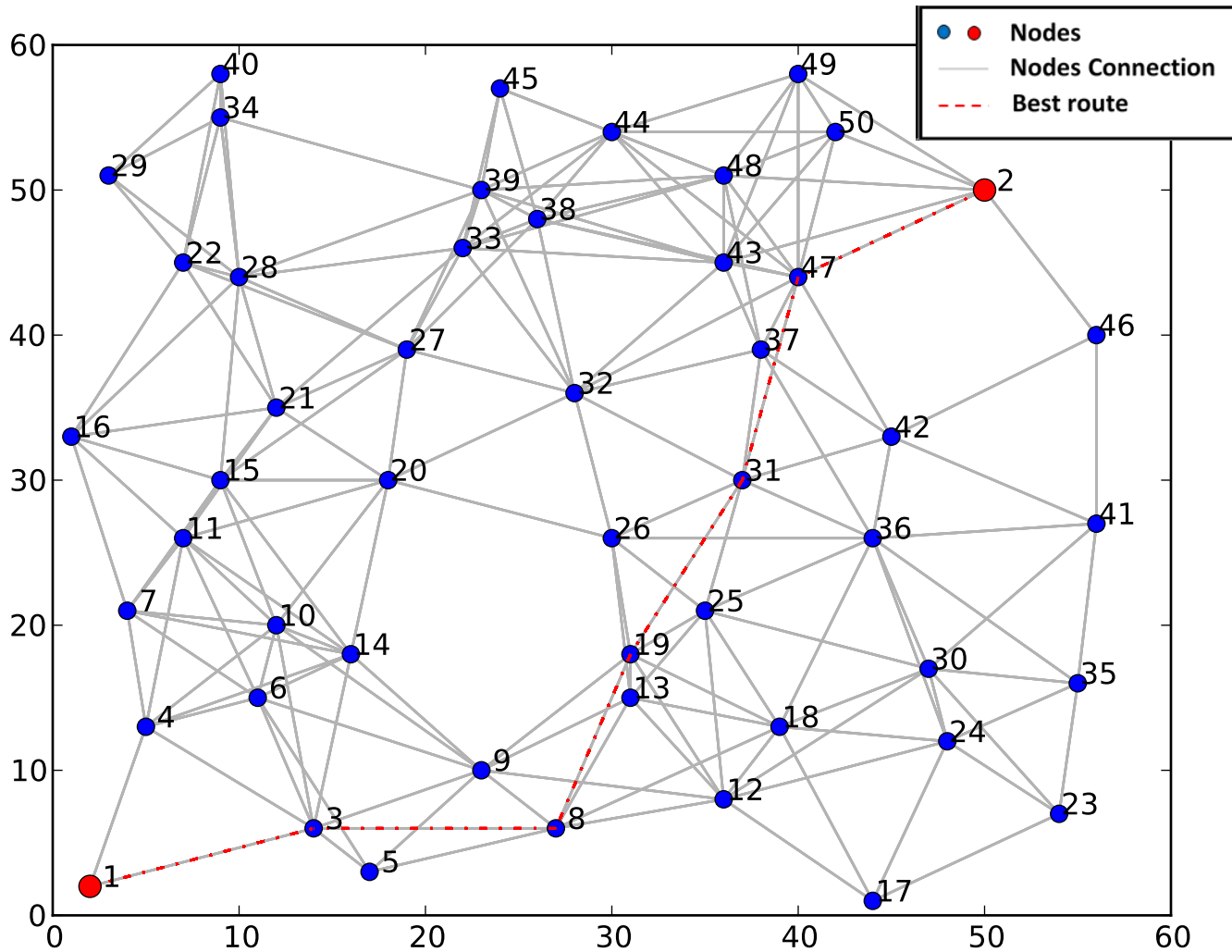

Supplement: Supplemental Information 1 — The code is written in NC programming language and is simulated using TOSSIM Simulator via Python script. The obtained values are available in the Excel files. [file peerj-cs-09-1682-s001.zip › Implementation/Simulation/Cost/results.pdf]

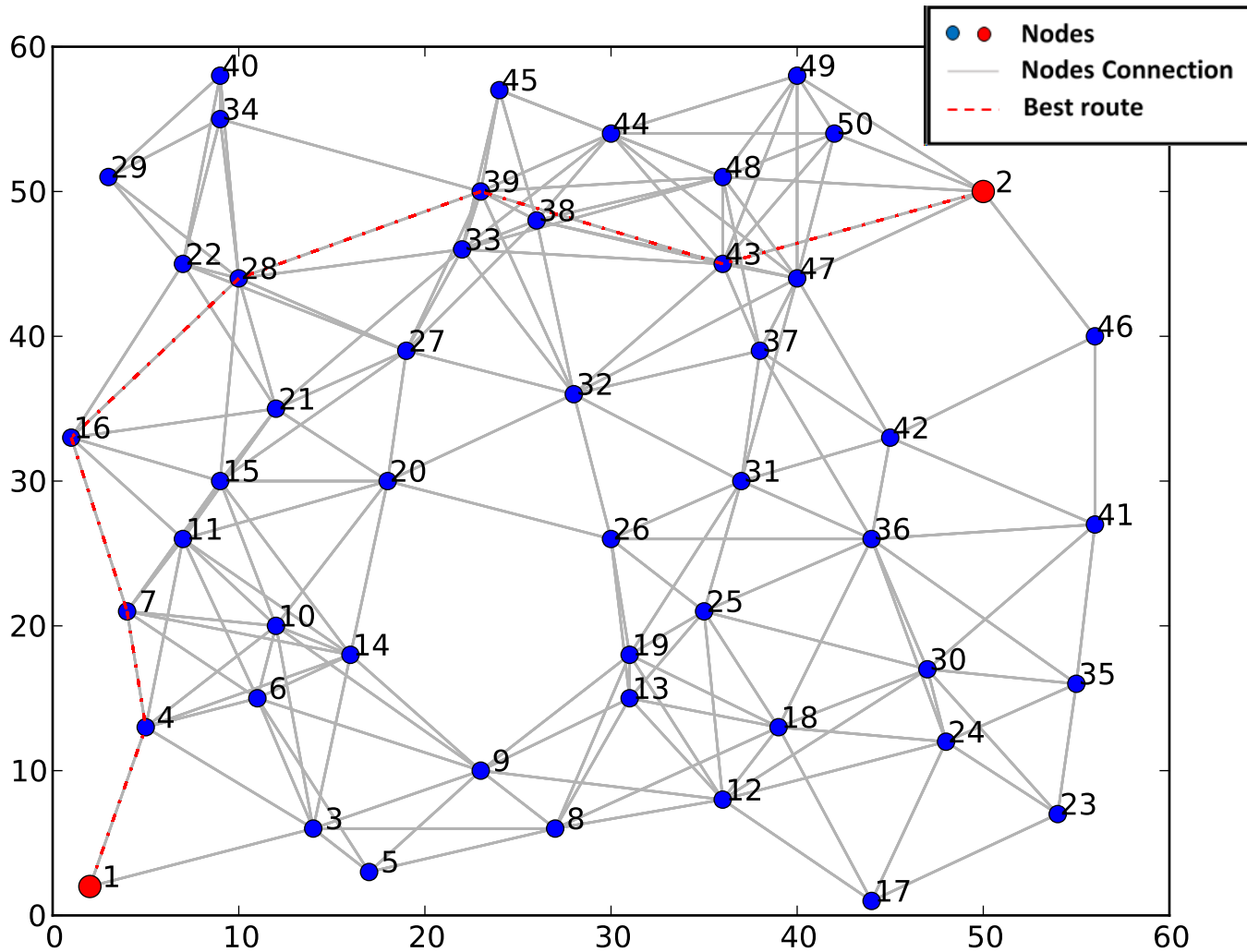

Supplement: Supplemental Information 1 — The code is written in NC programming language and is simulated using TOSSIM Simulator via Python script. The obtained values are available in the Excel files. [file peerj-cs-09-1682-s001.zip › Implementation/Simulation/CostDelayEnergy/results request 1.pdf]

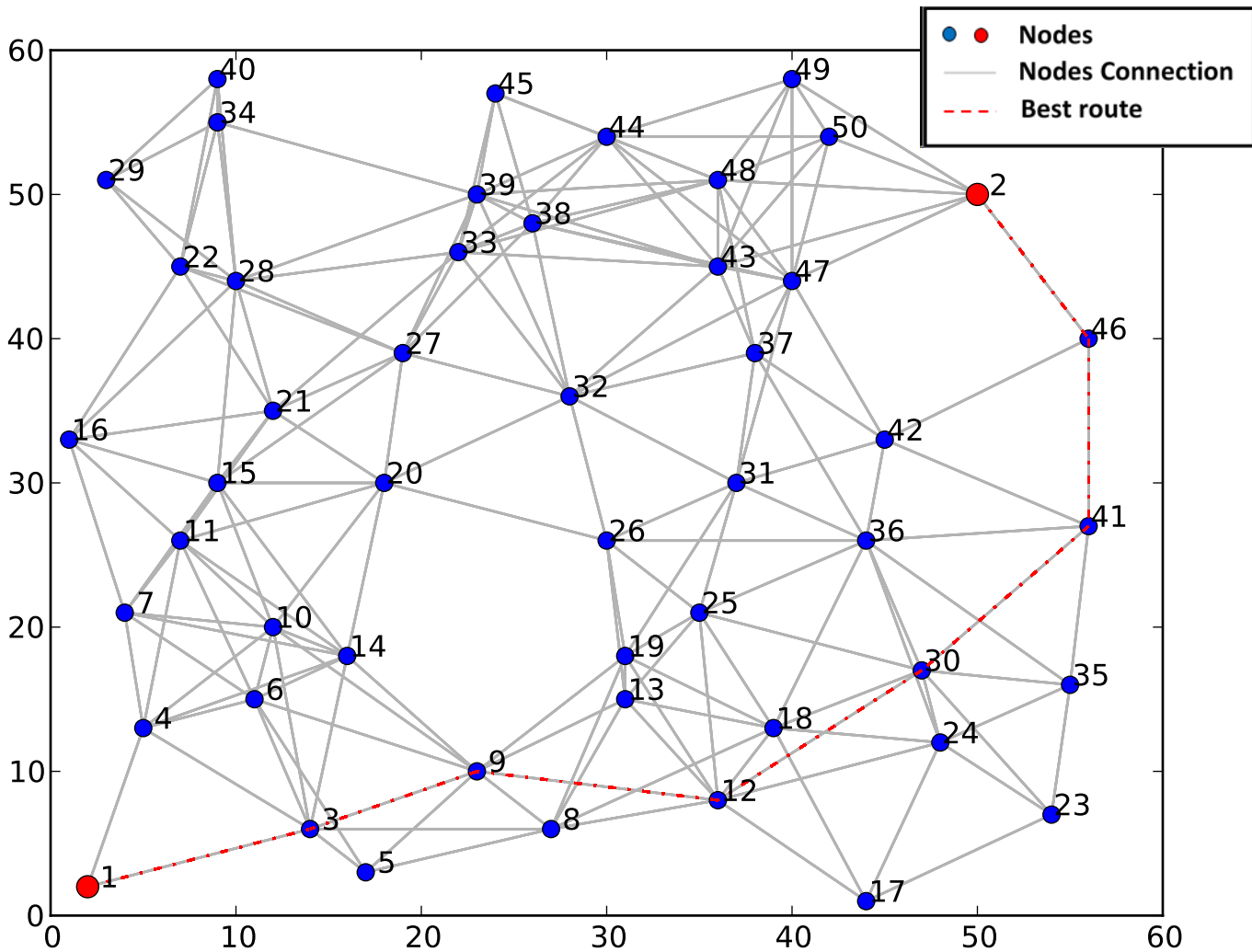

Supplement: Supplemental Information 1 — The code is written in NC programming language and is simulated using TOSSIM Simulator via Python script. The obtained values are available in the Excel files. [file peerj-cs-09-1682-s001.zip › Implementation/Simulation/CostDelayEnergy/results request 2.pdf]

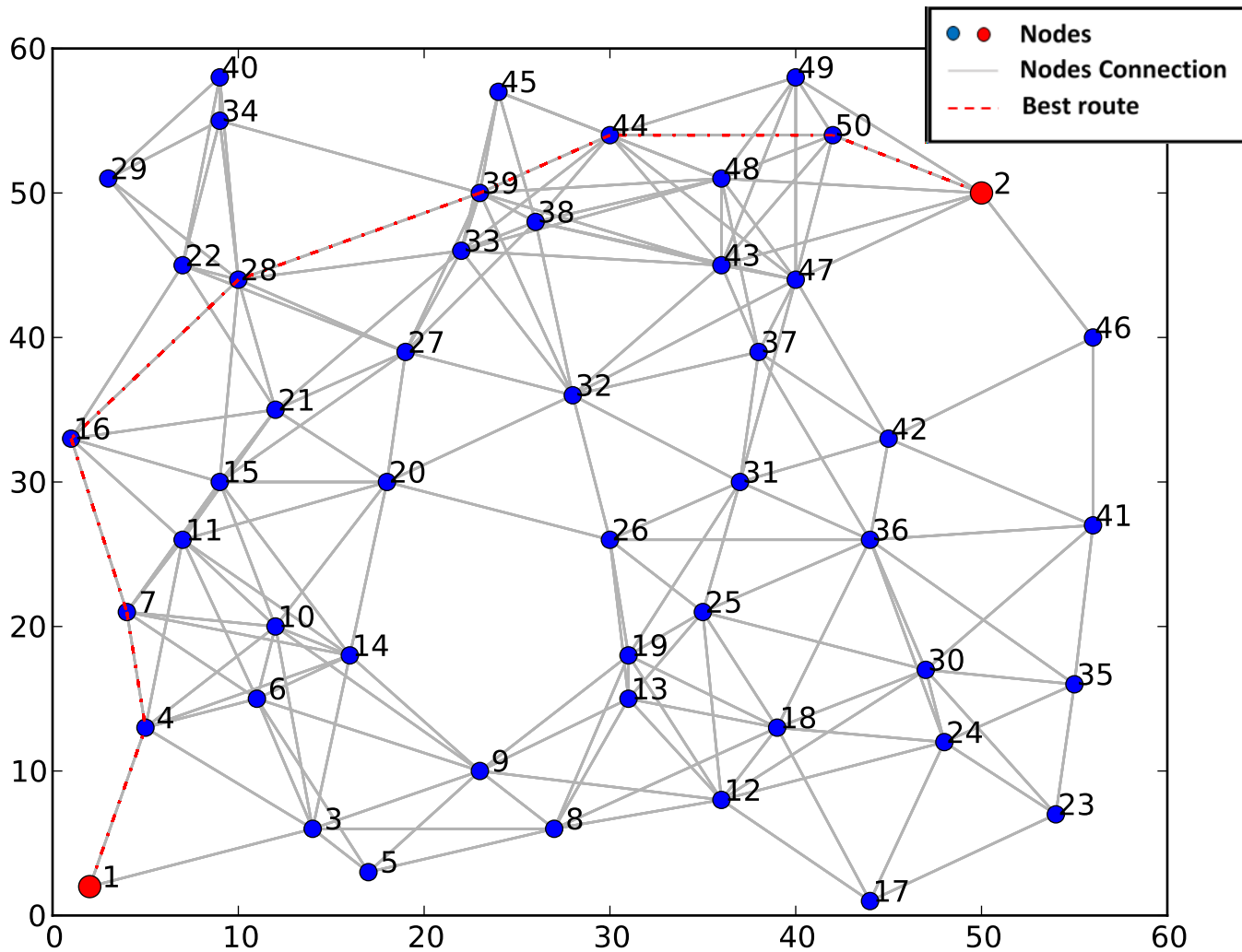

Supplement: Supplemental Information 1 — The code is written in NC programming language and is simulated using TOSSIM Simulator via Python script. The obtained values are available in the Excel files. [file peerj-cs-09-1682-s001.zip › Implementation/Simulation/Delay/results.pdf]

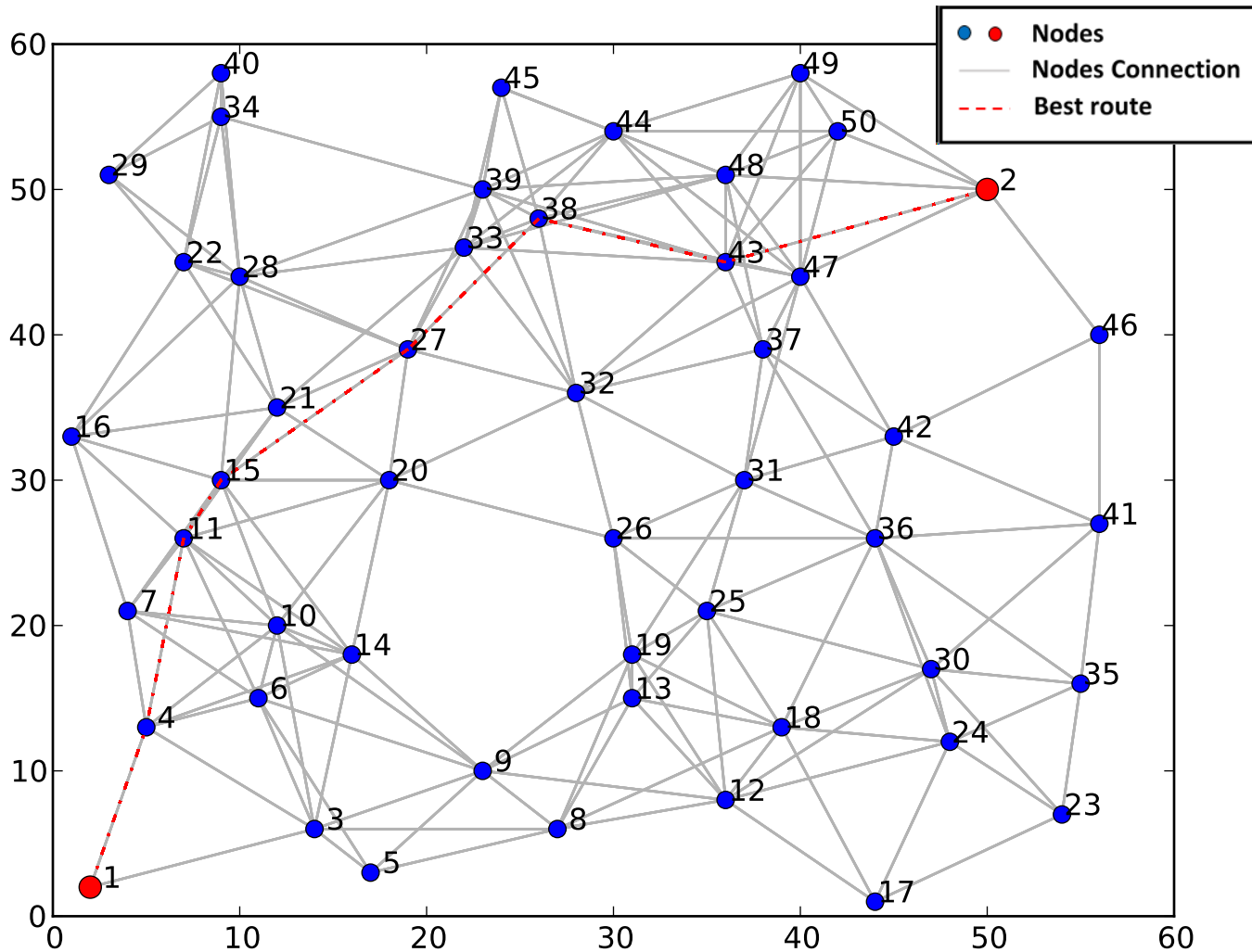

Supplement: Supplemental Information 1 — The code is written in NC programming language and is simulated using TOSSIM Simulator via Python script. The obtained values are available in the Excel files. [file peerj-cs-09-1682-s001.zip › Implementation/Simulation/CostDelai/results request 1.pdf]

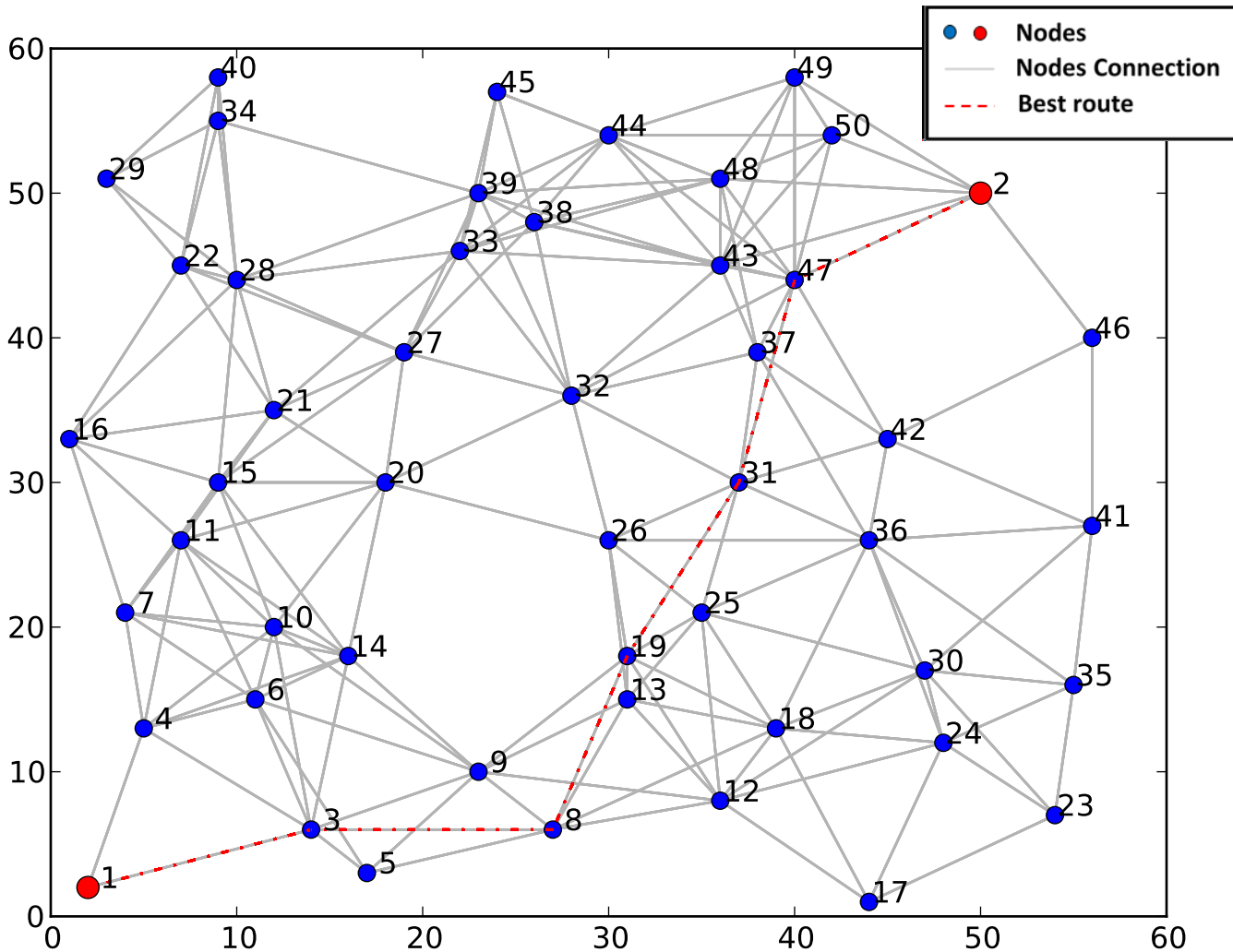

Supplement: Supplemental Information 1 — The code is written in NC programming language and is simulated using TOSSIM Simulator via Python script. The obtained values are available in the Excel files. [file peerj-cs-09-1682-s001.zip › Implementation/Simulation/CostDelai/results request 2.pdf]
